# Diagnostic yield and complication rate of transbronchial lung cryobiopsy using 1.1 mm probe in patients with interstitial lung disease

**DOI:** 10.1371/journal.pone.0354460

**Published:** 2026-07-30

**Authors:** Margaret A. Kypreos, Kim C. Styrvoky, Steven Wu, Audra Schwalk, Traci N. Adams

**Affiliations:** 1 Division of Pulmonary and Critical Care Medicine, University of Texas Southwestern Medical Center, Dallas, Texas, United States of America; 2 Section of Interventional Pulmonology, Division of Thoracic Oncology, Department of Surgical Oncology, Orlando Health Cancer Institute, Orlando, Florida, United States of America; University of Pittsburgh Medical Center Pinnacle Health Medical Services, UNITED STATES OF AMERICA

## Abstract

**Background:**

Widespread adoption of transbronchial lung cryobiopsy (TBLC) using a 1.9 or 2.4 mm touch cryoprobe in interstitial lung disease (ILD) has been limited by high bleeding risk. Recent studies have demonstrated that the 1.1 mm cryoprobe via conventional bronchoscope may achieve diagnostic yields comparable to larger probes while maintaining a favorable safety profile. Studies of the 1.1 mm touch cryoprobe in the diagnosis of ILD have been flawed and conflicting.

**Aim:**

The aim of this study is to describe the complication rate, diagnostic yield, and factors that increase diagnostic yield of TBLC using a 1.1 mm touch cryoprobe in patients with ILD.

**Methods:**

The retrospective cohort study included patients that were prospectively identified in the ILD clinic registry at University of Texas Southwestern Medical Center (UTSW) who underwent TBLC from 2022–2026. Procedural characteristics were recorded. Patients were assigned a pre-TBLC, post-TBLC, and final diagnosis.

**Results:**

This study demonstrates that TBLC using a 1.1 mm probe added to BAL significantly increases diagnostic yield with an 11% risk of pneumothorax and a 6.5% risk of bleeding. The combination of BAL and TBLC was diagnostic in 27 (43.5%) of patients and led to a change in diagnosis in 27 (43.5%) of patients. The number of TBLC samples taken is associated with a higher diagnostic yield.

**Conclusion:**

The diagnostic yield and complication rates of the 1.1 mm touch probe in our study are higher than previously reported studies with TBBx but lower than 1.9 and 2.4 mm touch probes, though there was no direct comparison group in our study. To increase yield of the procedure, multiple lobes should be biopsied and more samples taken.

## Introduction

Interstitial lung disease (ILD) is an umbrella term that encompasses more than 100 distinct subtypes. Classification of ILD into specific subtypes has implications for prognosis and treatment [1–3]. When the diagnosis of an ILD subtype cannot be determined noninvasively by assimilating clinical, serologic, and radiographic data, invasive procedures such as surgical lung biopsy (SLB) may be considered to assist in making a diagnosis [3–5]. SLB unfortunately has a high risk of complications, including a 1–2% mortality rate [6]; thus, minimally invasive techniques such as transbronchial lung cryobiopsy (TBLC) have been explored to obtain tissue for ILD diagnosis while minimizing the risk to patient safety [3,5,7,8].

While TBLC has a lower diagnostic yield (80%) compared to SLB (90%), TBLC is a less invasive and lower cost procedure than SLB [3]. Widespread adoption of TBLC has been limited by local expertise as well as complication rates, including a 9% risk of pneumothorax, 30% risk of any bleeding, and rare risk of major bleeding when a 2.4 mm or 1.9 mm TBLC probe is used [3,7,9]. TBLC for ILD has traditionally been performed via flexible bronchoscopy using these larger probes. Since the introduction of the 1.1 mm touch cryoprobe, it has primarily been utilized with conventional or navigational bronchoscopy for the evaluation of peripheral pulmonary lesions with the hope of reduced bleeding risk [10,11]. Recent studies have demonstrated that the 1.1 mm cryoprobe via conventional bronchoscope may achieve diagnostic yields comparable to larger probes while maintaining a favorable safety profile [12,13].

Robotic assisted bronchoscopy with shape-sensing technology (ssRAB), in combination with radial endobronchial ultrasound (r-EBUS), cone beam computed tomography (CBCT) and augmented fluoroscopy allows for precise targeting of parenchymal abnormalities [14]. Whether ssRAB with integration of multimodal imaging techniques with a 1.1 mm cryoprobe reduces complication rates or compromises diagnostic yield for the evaluation of ILD has not yet been determined. The purpose of the study is to explore the diagnostic yield and complication rates of a 1.1 mm TBLC probe with ssRAB, r-EBUS, CBCT, and augmented fluoroscopy for patients in the ILD clinic at a large academic medical center.

## Methods

The retrospective cohort study included patients that were prospectively identified in the ILD clinic registry at University of Texas Southwestern Medical Center (UTSW) who underwent TBLC from 2022−2026. Patients were excluded if they did not undergo TBLC during their diagnostic workup. This study was conducted in accordance with the amended Declaration of Helsinki and was approved by the UTSW Institutional Review Board (STU-2021–0346). Consent was not required due to the inability to obtain consent practically in this retrospective cohort study using a prospectively maintained registry and the use of de-identified data only. Ethnicity was self-reported by study subjects in the medical record, and subjects were not stratified or analyzed on the basis of confounding variables such as socioeconomic status or nutrition; this information was not available in the medical record and was not included. Data for this study was collected between January 2, 2025-February 28, 2026, though the procedures were completed between 2022 and 2026. Identifying data was not recorded or accessed during or after data collection.

Clinical data extracted from the medical record included age, gender, smoking history, causative antigen, results of pulmonary function testing (PFTs), high-resolution computed tomography (HRCT) results, BAL lymphocyte percentage, prior bronchoscopy results, TBLC results and complications, SLB results, and TBLC procedural characteristics, including number of samples, location of samples, probe size, anesthesia details, and procedure duration.

TBLC was performed using the ssRAB with the Ion robotic bronchoscopy platform (Intuitive Surgical, Sunnyvale, CA) under general anesthesia with endotracheal intubation. Pre-procedural CT chest imaging was uploaded into the Plan Point Software (Intuitive Surgical, Sunnyvale, CA) for procedural planning. Target sites were selected based on multidisciplinary discussion and radiographic features, preferentially targeting areas of ground glass opacity, nodularity, or less advanced fibrosis.

After airway inspection, the ssRAB was navigated to the planned target. Radial endobronchial ultrasound (r-EBUS) was used to confirm parenchymal localization and assess for adjacent vascular structures. Cone beam computed tomography (CBCT) imaging was performed using a Philips Allura Xper FD20 system (Philips Healthcare) with intra-procedural three-dimensional reconstruction and augmented fluoroscopic overlay used at the operator’s discretion to confirm catheter and/or cryoprobe positioning.

A 1.1-mm flexible cryoprobe (Erbe, Tübingen, Germany) was advanced under fluoroscopic guidance to approximately 1 cm from the pleural surface. Freeze times ranged from 4–6 seconds. The cryoprobe was removed following activation, and the specimen was retrieved and immediately placed in saline prior to fixation in formalin. Multiple biopsies were obtained per target site, with the number of samples and lobes biopsied determined by operator judgment and patient stability.

A bronchial blocker was not routinely used. Minor bleeding was defined as bleeding controlled with bronchoscopic suction, cold saline, and/or diluted epinephrine or tranexamic acid instillation as needed (Nashville Bleeding Grade 1–2). Major bleeding was defined as bleeding requiring balloon blocker placement, hemodynamic instability or unplanned procedure termination (Nashville Bleeding Grade 3–4) [15]. A post-procedure chest radiograph was obtained in all patients to evaluate for pneumothorax [14]. For the purposes of this study, each patient was assigned a multidisciplinary “pre-TBLC diagnosis” based on their clinical history, examination, serologic studies, and HRCT results without taking into account their bronchoalveolar lavage, TBLC, or SLB findings. A pre-TBLC diagnosis of HP was made if a patient met criteria for a moderately confident diagnosis of HP using current guidelines based on HRCT scan, antigen identification, and response to exposure removal (defined as a 10% improvement in forced vital capacity % predicted within 3 months or radiographic improvement on follow-up HRCT) [1,16]. A pre-TBLC diagnosis of IPF was made according to guidelines [3,4]. Patients meeting the American College of Rheumatology criteria for a defined connective tissue disease were assigned a pre-TBLC diagnosis of connective tissue disease-related ILD (CTD-ILD) [3]. Those who did not meet criteria for idiopathic pulmonary fibrosis (IPF), HP, CTD-ILD, or alternative cause of ILD were assigned a pre-TBLC diagnosis of unclassifiable ILD [3].

Next, patients were assigned a multidisciplinary “post-TBLC diagnosis” after incorporating their BAL and TBLC information in addition to the noninvasive testing. TBLC and BAL results were considered diagnostic based on current guidelines [1,3,4,16]. Lastly, patients were assigned a multidisciplinary “final diagnosis” utilizing all noninvasive testing, subsequent HRCT, SLB, and explant where available.

### Statistical analysis

Continuous variables were expressed as means and standard deviations, and comparisons were made using Student’s t test or Wilcoxon signed rank sum test as appropriate. Categorical variables were expressed using counts and percentages, and comparisons were made using Chi-squared test or Fisher’s exact test, where appropriate. Univariable logistic regression was performed to identify factors that were associated with diagnostic BAL or TBLC. These variables were chosen based on clinical relevance and included demographic characteristics, use of immunosuppression, whether the fibrogenic exposure had been removed prior to the bronchoscopy, number of lobes biopsied on TBLC, and HRCT findings in the locations of BAL and TBLC. The variables that were significantly associated with change in diagnosis (p-value <0.1) were included in multivariable model to test independent associations. All p-values less than 0.05 were considered significant. Statistical analyses were performed using *MedCalc* Statistical Software version 19.2.6 (*MedCalc* Software bv Ostend, Belgium; https://www.*medcalc*.org; 2020).

## Results

The study included 62 patients who underwent TBLC at UTSW for the diagnosis of ILD. Mean age was 66.2 (SD 11.8), and the majority were female (N = 37, 60%) and non-Hispanic white (N = 50, 81%) (Table 1). Cough and dyspnea were commonly present at the time of bronchoscopy. More than half of patients had crackles or rales on lung ausculatation. Over half had a fibrogenic antigen identified in their history. At baseline patients had a mild degree of lung impairment on PFTs. The majority of patients had fibrosis and ground glass on HRCT.

**Table 1:** Characteristics of patients with ILD who underwent TBLC (N = 62).

	ILD Patients undergoing TBLC (N = 62)
Mean age (SD)	66.2 (11.8)
Male, N (%)	25 (40.3)
Ethnicity, No. (%)	
Non-Hispanic White	50 (80.6)
Ever Smoker, N (%)	21 (33.9)
Symptoms, N (%)	
Cough	47 (75.8)
Dyspnea	53 (85.5)
Lung auscultation findings, N (%)	
Crackles and/or rales	38 (61.3)
Wheezing	3 (4.8)
Squeaks	5 (8.1)
Antigen identified, N (%)	36 (58.1)
In antigen exposure at time of TBLC	27 (43.5)
Clinical IPAF criteria, N (%)	45 (72.6)
Serologic IPAF criteria, N (%)	41 (66.1)
On immunosuppression at time of TBLC, N (%)	10 (16.1)
First degree relative with ILD, N (%)	8 (12.9)
Baseline Lung Function, mean (SD), N	
FVC % predicted	81 (17), 62
DLCO % predicted	63 (17), 62
Baseline supplemental oxygen use, N (%)	28 (45.1)
HRCT available for scoring, N (%)	62 (100%
Inconsistent with UIP	27 (43.5)
Indeterminate UIP	32 (51.6)
Probable UIP	2 (3.2)
Definite UIP	1 (1.6)
HRCT findings	
Fibrosis	49 (79.0)
Honeycombing	7 (11.3)
Ground glass	49 (79.0)
Air trapping	31 (50.0)
Micronodules	16 (25.8)
Consolidation	5 (8.1)
Invasive procedure Performed*, N (%)	
Surgical biopsy	2 (3.2)
TBBx	10 (16.1)
BAL	62 (100)
TBLC	62 (100)
ASA at time of procedure	
1	1 (1.2)
2	10 (20.7)
3	46 (72)
4	5 (6.1)
BMI, mean (SD) at time of procedure	30 (7.6)

* Some patients underwent multiple procedures.

TBLC was performed in 62 patients, sampling one lobe in 19 (30.6%), two lobes in 28 (45.2%), and three lobes in 14 (22.6%), with a mean of 5.0 ± 0.1 cryobiopsy specimens obtained per lobe. Mean total procedure time was 51.0 ± 11.6 minutes, including 34.0 ± 7.9 minutes of robotic navigation, and time to first tissue acquisition was 11.0 ± 5.3 minutes. Multimodal imaging was routinely used (radial EBUS 96.8%, fluoroscopy 100%, CBCT 96.8%), with significant body-to-CT divergence observed in 43.5% of cases. Mean fluoroscopy time was 2.5 ± 1.1 minutes, and mean cumulative air kerma and dose area product were 50.0 ± 26.4 mGy and 18.1 ± 9.8 Gy·cm^2^, respectively (Table 2).

**Table 2:** Procedure characteristics (N = 62).

	TBLC (N = 62)
**Procedural Details**	
Lobes biopsied, N (%)	
1 Lobe	19 (30.6)
2 Lobes	28 (45.2)
3 Lobes	14 (22.6)
# Cryo Biopsies per lobe, mean (SD)	5.0 (0.1)
Total procedure time, mean (SD)	51.0 (11.6) min
Total robotic time, mean (SD)	34.0 (7.9) min
Time from procedure start to first sample collection, mean (SD)	11.0 (5.3) min
**HRCT findings of biopsied segments**	
Fibrosis, N (%)	44 (70.1)
Ground glass, N (%)	42 (67.8)
Consolidation, N (%)	3 (4.8)
Micronodules, N (%)	15 (24.2)
Air trapping, N (%)	31 (50.0)
**Imaging Modalities**	
Radial EBUS used, N (%)	60 (96.8)
Fluoroscopy used, N (%)	62 (100)
Cone beam used, N (%)	60 (96.8)
Significant Body:CT Divergence, N (%)	27 (43.5)
CBCT, spins, mean (SD)	1.2 (0.5)
**Radiation Metrics**	
Total fluoro time, mean (SD)	2.5 (1.1) min
Cumulative CAK, mean (SD)	50.0 (26.4) mGy
Cumulative DAP, mean (SD)	18.1 (9.8) Gy·cm²
**Complications**	
Minor bleeding	4 (6.5)
Major bleeding	0 (0.0)
Pneumothorax	7 (11.3)
ILD exacerbation	1 (1.6)

Complications of TBLC included minor bleeding 4 (6.5%) treated with endobronchial epinephrine and/or cold saline instillation, major bleeding 0 (0%), pneumothorax 7 (11.3%), chest tube 5 (8.1%), and ILD exacerbation 1 (1.6%). Other reported outcomes post-TBLC included 1 (1.6%) hospital admission for altered mental status within a week of the procedure, 1 (1.6%) episode of hypoxemia treated with bilateral thoracentesis, and 1 (1.6%) complete heart block post-procedure requiring transvenous pacing. Four (6.5%) patients were inpatient at the time of the TBLC already; of the 58 outpatients who underwent TBLC, 8 (14.3%) required hospital admission post-procedure, including 1 (1.3%) ICU admission. No (0%) patients required re-intubation post-TBLC.

TBLC and BAL were diagnostic in 27 (43.5%) of patients; of patients with a diagnostic BAL and TBLC, all 27 (100%) had a change in diagnosis from pre-bronchoscopy to post-bronchoscopy.. Pre-bronchoscopy diagnoses included unclassifiable ILD in 56 patients (90.3%), HP in 5 (8.1%), and IPF 1 (1.6%). The most common post-bronchoscopy diagnoses included 29 (46.8%) with unclassifiable ILD, 16 (25.8%) with HP, 5 (8.1%) with COP, 3 (4.8%) with IPF, and 3 (4.8%) with sarcoidosis (Table 3). The final diagnosis in all 62 patients matched their post-bronchoscopy diagnosis because only 2 patients underwent SLB and in both cases their TBLC results were consistent with their SLB results, so SLB did not change the diagnosis.

**Table 3:** Pre-TBLC, post-TBLC, and final diagnosis (N = 62).

	Pre-TBLC Diagnosis N (%)*	Post-TBLC Diagnosis N (%)**	Final Diagnosis N (%)***
IPF	1 (1.6)	3 (4.8)	3 (4.8)
HP	5 (8.1)	16 (25.8)	16 (25.8)
Unclassifiable	56 (90.3)	29 (46.8)	29 (46.8)
Sarcoidosis	0 (0.0)	3 (4.8)	3 (4.8)
RB-ILD or DIP	0 (0.0)	1 (1.6)	1 (1.6)
Idiopathic NSIP	0 (0.0)	1 (1.6)	1 (1.6)
COP	0 (0.0)	5 (8.1)	5 (8.1)
Aspiration	0 (0.0)	1 (1.6)	1 (1.6)
GLILD	0 (0.0)	1 (1.6)	1 (1.6)
DIPNECH	0 (0.0)	1 (1.6)	1 (1.6)
ANCA vasculitis	0 (0.0)	1 (1.6)	1 (1.6)

*Pre-TBLC diagnosis takes into account the history, examination, serologic studies, and initial HRCT results.

**Post-TBLC diagnosis takes into account the history, examination, serologic studies, initial HRCT, and bronchoscopy results.

***Final diagnosis takes into account the history, examination, serologic studies, initial and subsequent HRCT, bronchoscopy, SLB, and explant results.

Abbreviations: IPF idiopathic pulmonary fibrosis; HP hypersensitivity pneumonitis; RB-ILD respiratory bronchiolitis interstitial lung disease; DIP desquamative interstitial pneumonia; NSIP nonspecific interstitial pneumonia; COP cryptogenic organizing pneumonia; GLILD granulomatous lymphocytic interstitial lung disease; DIPNECH diffuse idiopathic pulmonary neuroendocrine cell hyperplasia ANCA antineutrophic cytoplasmic antibody.

All patients who underwent TBLC also underwent BAL with lymphocyte percentage. TBLC when added to BAL increased the diagnostic yield of the procedure (7 [11.3%] patients achieved a diagnosis with BAL only compared to 27 [43.5%] with BAL+TBLC, p < 0.0001) (Table 4). Of the 16 patients with a final diagnosis of HP, diagnostic yield was higher for BAL + TBLC vs BAL alone (11 [68.8%] vs 2 [12.5%], p = 0.003).

**Table 4:** Comparison of diagnostic result with bronchoalveolar lavage to bronchoalveolar lavage + TBLC.

	BAL alone*	BAL and TBLC*	p-value
Overall (N = 62)	7	27	**< 0.001**
Final dx HP (N = 16)	2	11	**0.003**

*all patients who underwent TBLC also underwent BAL.

Factors predictive of a diagnostic TBLC in a univariable model included time from initial ILD clinic visit to procedure, significant CT:body divergence, presence of crackles or rales on physical exam, micronodules on HRCT in the segment where TBLC was done, fibrosis on HRCT in the segment where TBLC was done, number of lobes biopsied, number of cryo samples taken, and micronodules on HRCT (Table 5). Because micronodules anywhere on HRCT and micronodules on HRCT in the biopsied segment were co-linear, only micronodules on HRCT in the biopsied segment was included in the multivariable model due to the strength of the association; similarly, the number of lobes biopsied and number of TBLC samples were co-linear and the number of samples was chosen in the multivariable model due to the strength of the association. In a multivariable model including time from first ILD clinic visit to procedure, significant CT:body divergence, crackles or rales on physical exam, micronodules on HRCT in the biopsied segment, fibrosis on HRCT in the biopsied segment, and number of TBLC samples taken, only the number of TBLC samples taken (OR 1.1, 95% CI 1.0–1.2, p = 0.04) had a significant association with a change in diagnosis post-procedure.

**Table 5:** Factors predictive of a diagnostic TBLC.

Variable	Univariable model		Multivariable model	
	OR (95% CI)	P value	OR (95% CI)	P value
Antigen exposure	0.92 (0.3-2.5)	0.88		
Age	0.99 (0.9-1.0)	0.68		
Female Gender	0.57 (0.2-1.6)	0.35		
BMI	0.96 (0.88-1.0)	0.33		
Days from first ILD clinic visit to procedure	1.0 (0.99-1.0)	0.02	1.0 (0.99-1.0)	0.26
Cough	0.7 (0.4-1.2)	0.52		
Dyspnea	1.0 (0.2-4.3)	0.97		
Clinical IPAF criteria	1.6 (0.5-5.4)	0.54		
Serologic IPAF criteria	0.7 (0.2-2.0)	0.61		
On IS at time of bronch	2.2 (0.5-9.6)	0.33		
Ever smoker	0.7 (0.2-2.1)	0.61		
Antigen at time of bronch	1.33 (0.5-3.7)	0.64		
First degree relative with ILD	0.8 (0.1-3.4)	0.83		
Baseline supplemental oxygen use	0.4 (0.1-1.2)	0.16		
Crackles/rales	0.3 (0.1-0.9)	0.06	0.5 (0.1-2.5)	0.42
Squeaks	2.2 (0.3-17.3)	0.70		
HRCT fibrosis	0.4 (0.1-1.4)	0.30		
HRCT ground glass	0.4 (0.1-1.4)	0.30		
HRCT air trapping	1.3 (0.5-4.0)	0.51		
HRCT micronodules	6.2 (1.9-25.3)	0.03		
HRCT consolidation	2.1 (0.3-16.6)	0.72		
Baseline FVC % pred	0.8 (0.5-1.3)	1.0		
Baseline DLCO % pred	0.8 (0.4-1.3)	1.0		
Tidal volume during TBLC	1.0 (1.0-1.0)	0.58		
Time procedure to first sample	0.8 (0.8-1.2)	1.0		
Significant body:CT divergence	0.2 (0.1-0.7)	0.02	0.8 (0.2-4.0)	0.78
Number spins	1.0 (0.3-2.6)	0.81		
Number lobes biopsied	2.7 (1.3-6.1)	0.01		
HRCT GGO biopsy site	0.7 (0.2-1.8)	0.50		
HRCT micronodules biopsy site	9.7 (2.2-68.0)	0.04	1.7 (0.2-16.6)	0.60
HRCT fibrosis biopsy site	0.3 (0.1-0.8)	0.06	0.3 (0.1-1.9)	0.21
HRCT air trapping biopsy site	0.3 (0.8-3.4)	0.72		
Number cryo probe biopsies	1.1 (1.0-1.2)	0.005	1.1 (1.0-1.2)	0.04

## Discussion

This study demonstrates that TBLC using a 1.1 mm probe added to BAL significantly increases diagnostic yield with an 11% risk of pneumothorax and a 6.5% risk of bleeding. The combination of BAL and TBLC led to a change in diagnosis in 27 (43.5%) of patients. The number of TBLC samples taken is associated with a higher diagnostic yield.

The yield of TBLC in the present study is lower than prior studies [3,7–9,11,12,14]. The lower yield observed in the present study compared to prior 1.1 mm probe studies could be due to differences in bronchoscopic platform (ssRAB vs. conventional flexible bronchoscopy), patient population complexity (90.3% unclassifiable ILD vs. broader ILD cohorts), or the definition of diagnostic yield (change in diagnosis vs. MDD-confirmed diagnosis). Prior studies of 1.9 and 2.4 mm cryo probes have reported a yield between 60 and 80% [3,7–9,11,12,14,17–21]. Studies of 1.1 mm touch probes have > 80% diagnostic yield, which is much higher than expected, indicating the need for additional studies [12,13].

The present study demonstrates that the yield of a smaller probe is lower than the larger probes but still higher than TBBx, which has a yield of about 25% overall [22,23]. In contrast to TBBx under fluoroscopy, the integration of ssRAB with a 1.1 mm cryoprobe provides enhances distal airway navigation and positional stability, permitting targeted biopsy of peripheral parenchymal regions. Distal navigation of the robotic platform may also promote localized atelectasis by obstructing distal bronchus, potentially improving cryobiopsy specimen acquision. In addition, the adjunctive use of R-EBUS enhances procedural precision. Real-time fluoroscopy and ultrasound visualization allow estimation of pleural proximity and identification of adjacent vascular structures, which can help decrease clinically significant hemoptysis. An additional advantage of ssRAB is the ability to reproducibly target multiple lobes or segments within a single procedure without significant loss of navigational accuracy. This may be particularly relevant in interstitial lung disease, where diagnostic confidence can depend on sampling from multiple anatomic regions to allow more adequate tissue samples submitted. The platform’s stability allows for systematic, multi-lobar sampling while maintaining procedural efficiency. Despite significant CT-body divergence during the procedure, sampling area can still be optimized using pre-view path technique.

The rate of bleeding complications with the 1.1 mm cryoprobe are lower than previously reported studies of 1.9 mm and 2.4 mm cryoprobes, likely due to improved ability to maintain hemostasis with the smaller probes. The 1.1 mm robotic catheter itself may function as a mechanical tamponade device. When positioned within higher generation bronchus, the catheter can partially occlude the bronchial lumen, potentially limiting bleeding proximally into larger airways in the event of post-biopsy bleeding. This stable catheter position may therefore contribute to intrinsic hemostatic control without the routine need for adjunctive balloon blockers, which were not utilized in study.

Pneumothorax risk is comparable between cryoprobe sizes. Compared with larger cryoprobes, the smaller probe provides reduced tactile feedback during advancement. Diminished depth perception may increase the risk of pleural proximity at the time of activation, potentially contributing to pneumothorax.

Limitations of this study include retrospective cohort study of a prospectively maintained registry and absence of a control arm with larger probe sizes or SLB. Conclusions comparing diagnostic yield and complication rates of the 1.1 mm probe with larger cryoprobes should be interpreted more cautiously as there is no direct comparison group in this study. TBLC was ordered according to physician preference, and practices may not be generalizable across centers. Further, while our pre-TBLC diagnosis of HP was consistent with current guidelines, the diagnostic confidence of HP without BAL and TBLC may vary across centers or with future iterations of the guidelines, thus limiting the generalizability of the study. Sample sizes for the regression analysis of diagnostic features of TBLC were small, which may limit the strength of conclusions. Future multi-site studies directly comparing the yield of different size probes in the same patient may be conducted to validate our findings. Strengths of this study include multidisciplinary discussion, strict coding of diagnoses according to current guidelines, and procedural volume.

In conclusion, the diagnostic yield of the 1.1 mm touch probe in the diagnosis of ILD is 43.5%. However, complications include minor bleeding in 6.5% and pneumothorax in 11%. The diagnostic yield and complication rates of the 1.1 mm touch probe in our study are higher than previously reported studies with TBBx but lower than 1.9 and 2.4 mm touch probes, though there was no direct comparison group in our study. To increase yield of the procedure, multiple lobes should be biopsied and more samples taken.

## Supporting information

S1 Text Deidentified data. (XLSX)
