# Supplementary material for: Diagnostic yield and complication rate of transbronchial lung cryobiopsy using 1.1 mm probe in patients with interstitial lung disease
Source: PLoS One. 2026 Jul 30;21(7):e0354460. doi: 10.1371/journal.pone.0354460 (PMC13422831; doi:10.1371/journal.pone.0354460)
Supplement: Attachment — Submitted filename: Response to reviewers.docx [file pone.0354460.s003.docx]

**Reviewer #1: Thank you for submitting this manuscript. As mentioned in the manuscript, the main limitation of this study is the retrospective nature of the study and the lack of control arm. Despite reduced bleeding risk with 1.1 mm cryo probe compared to 1.9 mm and 2.4 mm probes the diagnostic yield is significantly reduced thus reducing the utility of the 1.1 mm probe particularly with only a rare risk of major bleeding with the larger probes. Also the smaller probe had slightly increased risk of pneumothorax. At this time I am not convinced that the smaller probe offers any significant advantages over the larger ones. Future prospective multisite study/studies comparing different size probes with a control arm is needed.**

We agree that future multi-site studies directly comparing the yield of different size probes in the same patient may be conducted to validate our findings and have included a statement to this effect in the manuscript.

**Reviewer #2: This manuscript evaluates the diagnostic yield and complication rate of transbronchial lung cryobiopsy using a 1.1 mm cryoprobe with robotic bronchoscopy guidance in patients undergoing evaluation for interstitial lung disease. The topic is clinically relevant, especially as smaller cryoprobes and robotic bronchoscopy are being used more frequently to improve safety while maintaining diagnostic yield. The study provides useful procedural details and complication data from a real-world academic center experience. However, several points need clarification. The study is described as both prospective and retrospective in different parts of the manuscript, and this should be clarified for consistency.**

We apologize for the confusion. This is a retrospective cohort study using a prospectively maintained registry. We have clarified this in the Methods section, which now states:

“The retrospective cohort study included patients that were prospectively identified in the ILD clinic registry at University of Texas Southwestern Medical Center (UTSW) who underwent TBLC from 2022-2026. Patients were excluded if they did not undergo TBLC during their diagnostic workup. This study was conducted in accordance with the amended Declaration of Helsinki and was approved by the UTSW Institutional Review Board (STU-2021-0346). Consent was not required due to the inability to obtain consent practically in this retrospective cohort study using a prospectively maintained registry and the use of de-identified data only.”

**The definition of diagnostic yield is not clearly explained and appears to be based on change in multidisciplinary diagnosis after BAL and TBLC rather than biopsy diagnostic adequacy alone, which is different from how diagnostic yield is usually reported in prior TBLC studies. This should be clarified.**

We have clarified that the 27 individuals with a change in diagnosis did have a diagnostic bronchoscopy.

**“**TBLC and BAL were diagnostic in 27 (43.5%) of patients; of patients with a diagnostic BAL and TBLC, all 27 (100%) had a change in diagnosis from pre-bronchoscopy to post-bronchoscopy.”

**In addition, the conclusions comparing diagnostic yield and complication rates of the 1.1 mm probe with larger cryoprobes should be interpreted more cautiously since there is no direct comparison group in this study.**

We have added a limitation in the discussion section: “Further, conclusions comparing diagnostic yield and complication rates of the 1.1 mm probe with larger cryoprobes should be interpreted more cautiously as there is no direct comparison group in this study.”

We have also changed the last paragraph of the discussion to be more cautious with our conclusions:

In conclusion, the diagnostic yield of the 1.1mm touch probe in the diagnosis of ILD is 43.5%. However, complications include minor bleeding in 6.5% and pneumothorax in 11%. The diagnostic yield and complication rates of the 1.1mm touch probe in our study are higher than previously reported studies with TBBx but lower than 1.9 and 2.4mm touch probes, though there was no direct comparison group in our study. To increase yield of the procedure, multiple lobes should be biopsied and more samples taken.

**The regression analysis exploring predictors of diagnostic yield is interesting, but the relatively small sample size may limit the strength of these conclusions.**

We have added a sentence to address this in the limitations section of the discussion**: “**Sample sizes for the regression analysis of diagnostic features of TBLC were small, which may limit the strength of conclusions.”

**Minor grammar and wording edits would also improve clarity in several sections of the manuscript.**

We have made grammar and wording edits throughout the manuscript to improve clarity. **Overall, this is a useful and clinically relevant study that adds practical experience with the 1.1 mm cryoprobe and robotic bronchoscopy platform. The manuscript would be suitable for publication after clarification of the study design, clearer definition of diagnostic yield, and moderation of some conclusions.**

We appreciate the reviewer comments.

**Reviewer #3: Reviewer Comment — Study Design Clarification:
There is an inconsistency in how the study design is described that needs to be addressed. The Methods section describes patients as "prospectively identified," yet the ethics statement refers to this as a "retrospective study" and notes that consent was waived on that basis. The authors likely conducted a retrospective analysis of a prospectively maintained registry, but this is not clearly stated. Additionally, the ethics section lists a data collection period of January 2025–February 2026, while the procedural period spans 2022–2026, with no explanation for the discrepancy. Please add one to two sentences clearly describing the study design and clarifying what each date range refers to. Consistent use of a single term, such as "retrospective cohort study using a prospectively maintained registry" throughout the abstract and Methods could resolve this.**

Our study is as the reviewer mentions a retrospective cohort study using a prospectively maintained registry. We have made the requested changes. We have also clarified that while the registry includes patients who had a TBLC between 2022 and 2026, we did not collect data until 2025-2026. The methods section now states**:**

**“**The retrospective cohort study included patients that were prospectively identified in the ILD clinic registry at University of Texas Southwestern Medical Center (UTSW) who underwent TBLC from 2022-2026. Patients were excluded if they did not undergo TBLC during their diagnostic workup. This study was conducted in accordance with the amended Declaration of Helsinki and was approved by the UTSW Institutional Review Board (STU-2021-0346). Consent was not required due to the inability to obtain consent practically in this retrospective cohort study using a prospectively maintained registry and the use of de-identified data only. Ethnicity was self-reported by study subjects in the medical record, and subjects were not stratified or analyzed on the basis of confounding variables such as socioeconomic status or nutrition; this information was not available in the medical record and was not included. Data for this study was collected between January 2, 2025-February 28, 2026, though the procedures were completed between 2022 and 2026.” **Reviewer Comment — Discussion: Yield Comparison with Prior Studies:
The discussion states that "studies of 1.1mm touch probes have >80% diagnostic yield, which is much higher than expected, indicating the need for additional studies." While the introduction appropriately notes that Bian et al. and Zhang et al. used conventional bronchoscopy, the discussion does not carry this context forward when comparing yields. The lower diagnostic yield in the present study (43.5%) compared to prior 1.1mm probe studies could be due to a few reasons that are not acknowledged at this point in the manuscript. I suggest the authors add a sentence explaining this, for example: "The lower yield observed in the present study compared to prior 1.1mm probe studies could be due to differences in bronchoscopic platform (ssRAB vs. conventional flexible bronchoscopy), patient population complexity (90.3% unclassifiable ILD vs. broader ILD cohorts), and definition of diagnostic yield (change in diagnosis vs. MDD-confirmed diagnosis), among other causes.**

We have added that statement in the discussion:

**“**The lower yield observed in the present study compared to prior 1.1mm probe studies could be due to differences in bronchoscopic platform (ssRAB vs. conventional flexible bronchoscopy), patient population complexity (90.3% unclassifiable ILD vs. broader ILD cohorts), or the definition of diagnostic yield (change in diagnosis vs. MDD-confirmed diagnosis).” **Reviewer Comment — Table 5 Formatting Errors:
A few OR values in Table 5 appear to have typographic errors. "Antigen exposure" shows OR 9.92 (0.3–2.5) and "Age" shows OR 9.99 (0.9–1.0), both not possible given their non-significant p-values and confidence intervals that cross 1.0. The "9." is likely a formatting artifact, with the true values being 0.92 and 0.99 respectively. Please verify and correct all OR values in Table 5 to ensure they are consistent with the reported p-values and confidence intervals.**

We have made the requested changes and have reviewed the OR values and made several other changes. **I appreciated the opportunity to review this manuscript and believe it makes a valuable contribution to the interventional pulmonology literature. With targeted revisions, this manuscript will be strengthened and ready for publication.**

We appreciate the reviewer comments.
