# Supplementary material for: Diagnostic yield and complication rate of transbronchial lung cryobiopsy using 1.1 mm probe in patients with interstitial lung disease
Source: PLoS One. 2026 Jul 30;21(7):e0354460. doi: 10.1371/journal.pone.0354460 (PMC13422831; doi:10.1371/journal.pone.0354460)
Supplement: Attachment — Submitted filename: R2 response to reviewers.docx [file pone.0354460.s004.docx]

Response to reviewers

**Reviewer #1: Thank you for submitting the revision. Respone to the reviewers comments is appreciated. I feel this manuscript will make a significant contribution to IP literature.**

We appreciate reviewer 1 comments.

**Reviewer #2: The authors have adequately addressed the major concerns raised during the prior review. The study design is now clearly described as a retrospective cohort study using a prospectively maintained registry, and the distinction between the procedure period and data collection period has been clarified.

The definition of diagnostic yield has also been clarified. The authors now explain that BAL and TBLC were considered diagnostic in 27 patients, and that all of these patients had a change in diagnosis after bronchoscopy.

The conclusions are now more appropriately cautious. The authors acknowledge that there was no direct comparison group using larger cryoprobes, surgical lung biopsy, or another control arm. This is important because the study cannot definitively prove superiority or noninferiority of the 1.1 mm probe compared with larger probes.

The added discussion about why the diagnostic yield in this study was lower than prior 1.1 mm cryoprobe studies is also useful. Differences in bronchoscopic platform, patient complexity, and definition of diagnostic yield are reasonable explanations.

The authors also corrected the apparent typographic errors in Table 5 and reviewed the odds ratios. This resolves a key presentation issue from the prior version.

Overall, this is a clinically relevant and useful study. It adds practical data on the diagnostic yield and complication rate of 1.1 mm transbronchial lung cryobiopsy using robotic bronchoscopy guidance in patients with ILD. The main limitations remain the retrospective design, small sample size, lack of a control group, and single-center experience, but these are now clearly acknowledged. The conclusions are supported by the data as revised. I believe the manuscript is suitable for publication.**

We appreciate reviewer 2 comments.

**Reviewer #3: - The pre-TBLC hypersensitivity pneumonitis (HP) threshold appears conservative but guideline-consistent. That is reasonable, but it likely contributes to the large apparent post-TBLC shift toward HP (since many patients with antigen exposure and suggestive features were initially classified as unclassifiable ILD until tissue sampling increased diagnostic confidence). I would encourage the authors to acknowledge that this could be an important limitation of the baseline classification scheme of HP and not imply that all post-TBLC diagnostic movement, especially in this category, reflects newly discovered disease.**

We have added the following statement to the limitations section of the discussion:

**“**Further, while our pre-TBLC diagnosis of HP was consistent with current guidelines, the diagnostic confidence of HP without BAL and TBLC may vary across centers or with future iterations of the guidelines, thus limiting the generalizability of the study.”

**- Typo TLBC was noted, please change that to TBLC**

We have corrected the typo
