# Supplementary material for: Diagnostic yield and complication rate of transbronchial lung cryobiopsy using 1.1 mm probe in patients with interstitial lung disease
Source: PLoS One. 2026 Jul 30;21(7):e0354460. doi: 10.1371/journal.pone.0354460 (PMC13422831; doi:10.1371/journal.pone.0354460)
Supplement: Peer review history

# Peer review: Decision Letter 0

**DOI:** 10.1371/journal.pone.0354460.r001

**Authors:** Kartikeya Rajdev

28 Apr 2026

PONE-D-26-14642

Diagnostic yield and complication rate of transbronchial lung cryobiopsy using 1.1mm probe in patients with interstitial lung disease

PLOS One

Dear Dr. Adams,

Thank you for submitting your manuscript to PLOS ONE. After careful consideration, we feel that it has merit but does not fully meet PLOS ONE’s publication criteria as it currently stands. Therefore, we invite you to submit a revised version of the manuscript that addresses the points raised during the review process.

Please submit your revised manuscript by Jun 12 2026 11:59PM. If you will need more time than this to complete your revisions, please reply to this message or contact the journal office at plosone@plos.org. When you're ready to submit your revision, log on to https://www.editorialmanager.com/pone/ and select the 'Submissions Needing Revision' folder to locate your manuscript file.

Please include the following items when submitting your revised manuscript:

- A letter that responds to each point raised by the academic editor and reviewer(s). You should upload this letter as a separate file labeled 'Response to Reviewers'.
- A marked-up copy of your manuscript that highlights changes made to the original version. You should upload this as a separate file labeled 'Revised Manuscript with Track Changes'.
- An unmarked version of your revised paper without tracked changes. You should upload this as a separate file labeled 'Manuscript'.

If you would like to make changes to your financial disclosure, please include your updated statement in your cover letter. Guidelines for resubmitting your figure files are available below the reviewer comments at the end of this letter.

If applicable, we recommend that you deposit your laboratory protocols in protocols.io to enhance the reproducibility of your results. Protocols.io assigns your protocol its own identifier (DOI) so that it can be cited independently in the future. For instructions see: https://journals.plos.org/plosone/s/submission-guidelines#loc-laboratory-protocols. Additionally, PLOS ONE offers an option for publishing peer-reviewed Lab Protocol articles, which describe protocols hosted on protocols.io. Read more information on sharing protocols at https://plos.org/protocols?utm_medium=editorial-email&utm_source=authorletters&utm_campaign=protocols.

As the corresponding author, your ORCID iD is verified in the submission system and will appear in the published article. PLOS supports the use of ORCID, and we encourage all coauthors to register for an ORCID iD and use it as well. Please encourage your coauthors to verify their ORCID iD within the submission system before final acceptance, as unverified ORCID iDs will not appear in the published article. *Only*  the individual author can complete the verification step; PLOS staff *cannot*  verify ORCID iDs on behalf of authors.

We look forward to receiving your revised manuscript.

Kind regards,

Kartikeya Rajdev, MD

Academic Editor

PLOS One

Journal Requirements:

When submitting your revision, we need you to address these additional requirements.

1.Please ensure that your manuscript meets PLOS ONE's style requirements, including those for file naming. The PLOS ONE style templates can be found at

https://journals.plos.org/plosone/s/file?id=wjVg/PLOSOne_formatting_sample_main_body.pdf and

https://journals.plos.org/plosone/s/file?id=ba62/PLOSOne_formatting_sample_title_authors_affiliations.pdf

2. Please amend your manuscript to include your abstract after the title page.

3. We note you have included a table to which you do not refer in the text of your manuscript. Please ensure that you refer to Table 3 and 4 in your text; if accepted, production will need this reference to link the reader to the Table.

4. Please include captions for your Supporting Information files at the end of your manuscript, and update any in-text citations to match accordingly. Please see our Supporting Information guidelines for more information: http://journals.plos.org/plosone/s/supporting-information.

5. We note that there is identifying data in the Supporting Information file “Deidentified data cryo project.xlsx”. Due to the inclusion of these potentially identifying data, we have removed this file from your file inventory. Prior to sharing human research participant data, authors should consult with an ethics committee to ensure data are shared in accordance with participant consent and all applicable local laws.

Data sharing should never compromise participant privacy. It is therefore not appropriate to publicly share personally identifiable data on human research participants. The following are examples of data that should not be shared:

-Name, initials, physical address

-Ages more specific than whole numbers

-Internet protocol (IP) address

-Specific dates (birth dates, death dates, examination dates, etc.)

-Contact information such as phone number or email address

-Location data

-ID numbers that seem specific (long numbers, include initials, titled “Hospital ID”) rather than random (small numbers in numerical order)

Data that are not directly identifying may also be inappropriate to share, as in combination they can become identifying. For example, data collected from a small group of participants, vulnerable populations, or private groups should not be shared if they involve indirect identifiers (such as sex, ethnicity, location, etc.) that may risk the identification of study participants.

Additional guidance on preparing raw data for publication can be found in our Data Policy (https://journals.plos.org/plosone/s/data-availability#loc-human-research-participant-data-and-other-sensitive-data) and in the following article: http://www.bmj.com/content/340/bmj.c181.long.

Please remove or anonymize all personal information (<specific identifying information in file to be removed>), ensure that the data shared are in accordance with participant consent, and re-upload a fully anonymized data set. Please note that spreadsheet columns with personal information must be removed and not hidden as all hidden columns will appear in the published file.

6. If the reviewer comments include a recommendation to cite specific previously published works, please review and evaluate these publications to determine whether they are relevant and should be cited. There is no requirement to cite these works unless the editor has indicated otherwise.

7. Please review your reference list to ensure that it is complete and correct. If you have cited papers that have been retracted, please include the rationale for doing so in the manuscript text, or remove these references and replace them with relevant current references. Any changes to the reference list should be mentioned in the rebuttal letter that accompanies your revised manuscript. If you need to cite a retracted article, indicate the article’s retracted status in the References list and also include a citation and full reference for the retraction notice.

[Note: HTML markup is below. Please do not edit.]

Reviewers' comments:

Reviewer's Responses to Questions

**Comments to the Author**

1. Is the manuscript technically sound, and do the data support the conclusions?

The manuscript must describe a technically sound piece of scientific research with data that supports the conclusions. Experiments must have been conducted rigorously, with appropriate controls, replication, and sample sizes. The conclusions must be drawn appropriately based on the data presented.

Reviewer #1: Yes

Reviewer #2: Yes

Reviewer #3: Yes

**********

2. Has the statistical analysis been performed appropriately and rigorously?

Reviewer #1: Yes

Reviewer #2: Yes

Reviewer #3: Yes

**********

3. Have the authors made all data underlying the findings in their manuscript fully available?

The PLOS Data policy requires authors to make all data underlying the findings described in their manuscript fully available without restriction, with rare exception (please refer to the Data Availability Statement in the manuscript PDF file). The data should be provided as part of the manuscript or its supporting information, or deposited to a public repository. For example, in addition to summary statistics, the data points behind means, medians and variance measures should be available. If there are restrictions on publicly sharing data—e.g. participant privacy or use of data from a third party—those must be specified.

Reviewer #1: Yes

Reviewer #2: Yes

Reviewer #3: Yes

**********

4. Is the manuscript presented in an intelligible fashion and written in standard English?

PLOS ONE does not copyedit accepted manuscripts, so the language in submitted articles must be clear, correct, and unambiguous. Any typographical or grammatical errors should be corrected at revision, so please note any specific errors here.

Reviewer #1: Yes

Reviewer #2: Yes

Reviewer #3: Yes

**********

5. Review Comments to the Author

Please use the space provided to explain your answers to the questions above. You may also include additional comments for the author, including concerns about dual publication, research ethics, or publication ethics. (Please upload your review as an attachment if it exceeds 20,000 characters)

Reviewer #1: Thank you for submitting this manuscript. As mentioned in the manuscript, the main limitation of this study is the retrospective nature of the study and the lack of control arm. Despite reduced bleeding risk with 1.1 mm cryo probe compared to 1.9 mm and 2.4 mm probes the diagnostic yield is significantly reduced thus reducing the utility of the 1.1 mm probe particularly with only a rare risk of major bleeding with the larger probes. Also the smaller probe had slightly increased risk of pneumothorax. At this time I am not convinced that the smaller probe offers any significant advantages over the larger ones. Future prospective multisite study/studies comparing different size probes with a control arm is needed.

Reviewer #2: This manuscript evaluates the diagnostic yield and complication rate of transbronchial lung cryobiopsy using a 1.1 mm cryoprobe with robotic bronchoscopy guidance in patients undergoing evaluation for interstitial lung disease. The topic is clinically relevant, especially as smaller cryoprobes and robotic bronchoscopy are being used more frequently to improve safety while maintaining diagnostic yield. The study provides useful procedural details and complication data from a real-world academic center experience.

However, several points need clarification. The study is described as both prospective and retrospective in different parts of the manuscript, and this should be clarified for consistency. The definition of diagnostic yield is not clearly explained and appears to be based on change in multidisciplinary diagnosis after BAL and TBLC rather than biopsy diagnostic adequacy alone, which is different from how diagnostic yield is usually reported in prior TBLC studies. This should be clarified. In addition, the conclusions comparing diagnostic yield and complication rates of the 1.1 mm probe with larger cryoprobes should be interpreted more cautiously since there is no direct comparison group in this study. The regression analysis exploring predictors of diagnostic yield is interesting, but the relatively small sample size may limit the strength of these conclusions. Minor grammar and wording edits would also improve clarity in several sections of the manuscript.

Overall, this is a useful and clinically relevant study that adds practical experience with the 1.1 mm cryoprobe and robotic bronchoscopy platform. The manuscript would be suitable for publication after clarification of the study design, clearer definition of diagnostic yield, and moderation of some conclusions.

Reviewer #3: Reviewer Comment — Study Design Clarification:

There is an inconsistency in how the study design is described that needs to be addressed. The Methods section describes patients as "prospectively identified," yet the ethics statement refers to this as a "retrospective study" and notes that consent was waived on that basis. The authors likely conducted a retrospective analysis of a prospectively maintained registry, but this is not clearly stated. Additionally, the ethics section lists a data collection period of January 2025–February 2026, while the procedural period spans 2022–2026, with no explanation for the discrepancy. Please add one to two sentences clearly describing the study design and clarifying what each date range refers to. Consistent use of a single term, such as "retrospective cohort study using a prospectively maintained registry" throughout the abstract and Methods could resolve this.

Reviewer Comment — Discussion: Yield Comparison with Prior Studies:

The discussion states that "studies of 1.1mm touch probes have >80% diagnostic yield, which is much higher than expected, indicating the need for additional studies." While the introduction appropriately notes that Bian et al. and Zhang et al. used conventional bronchoscopy, the discussion does not carry this context forward when comparing yields. The lower diagnostic yield in the present study (43.5%) compared to prior 1.1mm probe studies could be due to a few reasons that are not acknowledged at this point in the manuscript. I suggest the authors add a sentence explaining this, for example: "The lower yield observed in the present study compared to prior 1.1mm probe studies could be due to differences in bronchoscopic platform (ssRAB vs. conventional flexible bronchoscopy), patient population complexity (90.3% unclassifiable ILD vs. broader ILD cohorts), and definition of diagnostic yield (change in diagnosis vs. MDD-confirmed diagnosis), among other causes.

Reviewer Comment — Table 5 Formatting Errors:

A few OR values in Table 5 appear to have typographic errors. "Antigen exposure" shows OR 9.92 (0.3–2.5) and "Age" shows OR 9.99 (0.9–1.0), both not possible given their non-significant p-values and confidence intervals that cross 1.0. The "9." is likely a formatting artifact, with the true values being 0.92 and 0.99 respectively. Please verify and correct all OR values in Table 5 to ensure they are consistent with the reported p-values and confidence intervals.

I appreciated the opportunity to review this manuscript and believe it makes a valuable contribution to the interventional pulmonology literature. With targeted revisions, this manuscript will be strengthened and ready for publication.

**********

6. PLOS authors have the option to publish the peer review history of their article (what does this mean?). If published, this will include your full peer review and any attached files.

If you choose “no”, your identity will remain anonymous but your review may still be made public.

**Do you want your identity to be public for this peer review?**  For information about this choice, including consent withdrawal, please see our Privacy Policy.

Reviewer #1: **Yes:** Shais Jallu

Reviewer #2: **Yes:** Abhishek Singh

Reviewer #3: **Yes:** Narayana Gandham

**********

[NOTE: If reviewer comments were submitted as an attachment file, they will be attached to this email and accessible via the submission site. Please log into your account, locate the manuscript record, and check for the action link "View Attachments". If this link does not appear, there are no attachment files.]

To ensure your figures meet our technical requirements, please review our figure guidelines: https://journals.plos.org/plosone/s/figures

You may also use PLOS’s free figure tool, NAAS, to help you prepare publication quality figures: https://journals.plos.org/plosone/s/figures#loc-tools-for-figure-preparation.

NAAS will assess whether your figures meet our technical requirements by comparing each figure against our figure specifications.

# Author comment: Author response to Decision Letter 1

**DOI:** 10.1371/journal.pone.0354460.r002

30 Apr 2026

Reviewer #1: Thank you for submitting this manuscript. As mentioned in the manuscript, the main limitation of this study is the retrospective nature of the study and the lack of control arm. Despite reduced bleeding risk with 1.1 mm cryo probe compared to 1.9 mm and 2.4 mm probes the diagnostic yield is significantly reduced thus reducing the utility of the 1.1 mm probe particularly with only a rare risk of major bleeding with the larger probes. Also the smaller probe had slightly increased risk of pneumothorax. At this time I am not convinced that the smaller probe offers any significant advantages over the larger ones. Future prospective multisite study/studies comparing different size probes with a control arm is needed.

We agree that future multi-site studies directly comparing the yield of different size probes in the same patient may be conducted to validate our findings and have included a statement to this effect in the manuscript.

Reviewer #2: This manuscript evaluates the diagnostic yield and complication rate of transbronchial lung cryobiopsy using a 1.1 mm cryoprobe with robotic bronchoscopy guidance in patients undergoing evaluation for interstitial lung disease. The topic is clinically relevant, especially as smaller cryoprobes and robotic bronchoscopy are being used more frequently to improve safety while maintaining diagnostic yield. The study provides useful procedural details and complication data from a real-world academic center experience. However, several points need clarification. The study is described as both prospective and retrospective in different parts of the manuscript, and this should be clarified for consistency.

We apologize for the confusion. This is a retrospective cohort study using a prospectively maintained registry. We have clarified this in the Methods section, which now states:

“The retrospective cohort study included patients that were prospectively identified in the ILD clinic registry at University of Texas Southwestern Medical Center (UTSW) who underwent TBLC from 2022-2026. Patients were excluded if they did not undergo TBLC during their diagnostic workup. This study was conducted in accordance with the amended Declaration of Helsinki and was approved by the UTSW Institutional Review Board (STU-2021-0346). Consent was not required due to the inability to obtain consent practically in this retrospective cohort study using a prospectively maintained registry and the use of de-identified data only.”

The definition of diagnostic yield is not clearly explained and appears to be based on change in multidisciplinary diagnosis after BAL and TBLC rather than biopsy diagnostic adequacy alone, which is different from how diagnostic yield is usually reported in prior TBLC studies. This should be clarified.

We have clarified that the 27 individuals with a change in diagnosis did have a diagnostic bronchoscopy.

“TBLC and BAL were diagnostic in 27 (43.5%) of patients; of patients with a diagnostic BAL and TBLC, all 27 (100%) had a change in diagnosis from pre-bronchoscopy to post-bronchoscopy.”

In addition, the conclusions comparing diagnostic yield and complication rates of the 1.1 mm probe with larger cryoprobes should be interpreted more cautiously since there is no direct comparison group in this study.

We have added a limitation in the discussion section: “Further, conclusions comparing diagnostic yield and complication rates of the 1.1 mm probe with larger cryoprobes should be interpreted more cautiously as there is no direct comparison group in this study.”

We have also changed the last paragraph of the discussion to be more cautious with our conclusions:

In conclusion, the diagnostic yield of the 1.1mm touch probe in the diagnosis of ILD is 43.5%. However, complications include minor bleeding in 6.5% and pneumothorax in 11%. The diagnostic yield and complication rates of the 1.1mm touch probe in our study are higher than previously reported studies with TBBx but lower than 1.9 and 2.4mm touch probes, though there was no direct comparison group in our study. To increase yield of the procedure, multiple lobes should be biopsied and more samples taken.

The regression analysis exploring predictors of diagnostic yield is interesting, but the relatively small sample size may limit the strength of these conclusions.

We have added a sentence to address this in the limitations section of the discussion: “Sample sizes for the regression analysis of diagnostic features of TBLC were small, which may limit the strength of conclusions.”

Minor grammar and wording edits would also improve clarity in several sections of the manuscript.

We have made grammar and wording edits throughout the manuscript to improve clarity.

Overall, this is a useful and clinically relevant study that adds practical experience with the 1.1 mm cryoprobe and robotic bronchoscopy platform. The manuscript would be suitable for publication after clarification of the study design, clearer definition of diagnostic yield, and moderation of some conclusions.

We appreciate the reviewer comments.

Reviewer #3: Reviewer Comment — Study Design Clarification:

There is an inconsistency in how the study design is described that needs to be addressed. The Methods section describes patients as "prospectively identified," yet the ethics statement refers to this as a "retrospective study" and notes that consent was waived on that basis. The authors likely conducted a retrospective analysis of a prospectively maintained registry, but this is not clearly stated. Additionally, the ethics section lists a data collection period of January 2025–February 2026, while the procedural period spans 2022–2026, with no explanation for the discrepancy. Please add one to two sentences clearly describing the study design and clarifying what each date range refers to. Consistent use of a single term, such as "retrospective cohort study using a prospectively maintained registry" throughout the abstract and Methods could resolve this.

Our study is as the reviewer mentions a retrospective cohort study using a prospectively maintained registry. We have made the requested changes. We have also clarified that while the registry includes patients who had a TBLC between 2022 and 2026, we did not collect data until 2025-2026. The methods section now states:

“The retrospective cohort study included patients that were prospectively identified in the ILD clinic registry at University of Texas Southwestern Medical Center (UTSW) who underwent TBLC from 2022-2026. Patients were excluded if they did not undergo TBLC during their diagnostic workup. This study was conducted in accordance with the amended Declaration of Helsinki and was approved by the UTSW Institutional Review Board (STU-2021-0346). Consent was not required due to the inability to obtain consent practically in this retrospective cohort study using a prospectively maintained registry and the use of de-identified data only. Ethnicity was self-reported by study subjects in the medical record, and subjects were not stratified or analyzed on the basis of confounding variables such as socioeconomic status or nutrition; this information was not available in the medical record and was not included. Data for this study was collected between January 2, 2025-February 28, 2026, though the procedures were completed between 2022 and 2026.”

Reviewer Comment — Discussion: Yield Comparison with Prior Studies:

The discussion states that "studies of 1.1mm touch probes have >80% diagnostic yield, which is much higher than expected, indicating the need for additional studies." While the introduction appropriately notes that Bian et al. and Zhang et al. used conventional bronchoscopy, the discussion does not carry this context forward when comparing yields. The lower diagnostic yield in the present study (43.5%) compared to prior 1.1mm probe studies could be due to a few reasons that are not acknowledged at this point in the manuscript. I suggest the authors add a sentence explaining this, for example: "The lower yield observed in the present study compared to prior 1.1mm probe studies could be due to differences in bronchoscopic platform (ssRAB vs. conventional flexible bronchoscopy), patient population complexity (90.3% unclassifiable ILD vs. broader ILD cohorts), and definition of diagnostic yield (change in diagnosis vs. MDD-confirmed diagnosis), among other causes.

We have added that statement in the discussion:

“The lower yield observed in the present study compared to prior 1.1mm probe studies could be due to differences in bronchoscopic platform (ssRAB vs. conventional flexible bronchoscopy), patient population complexity (90.3% unclassifiable ILD vs. broader ILD cohorts), or the definition of diagnostic yield (change in diagnosis vs. MDD-confirmed diagnosis).”

Reviewer Comment — Table 5 Formatting Errors:

A few OR values in Table 5 appear to have typographic errors. "Antigen exposure" shows OR 9.92 (0.3–2.5) and "Age" shows OR 9.99 (0.9–1.0), both not possible given their non-significant p-values and confidence intervals that cross 1.0. The "9." is likely a formatting artifact, with the true values being 0.92 and 0.99 respectively. Please verify and correct all OR values in Table 5 to ensure they are consistent with the reported p-values and confidence intervals.

We have made the requested changes and have reviewed the OR values and made several other changes.

I appreciated the opportunity to review this manuscript and believe it makes a valuable contribution to the interventional pulmonology literature. With targeted revisions, this manuscript will be strengthened and ready for publication.

We appreciate the reviewer comments.

Attachment Submitted filename: Response to reviewers.docx

# Peer review: Decision Letter 1

**DOI:** 10.1371/journal.pone.0354460.r003

**Authors:** Kartikeya Rajdev

17 Jun 2026

PONE-D-26-14642R1

Diagnostic yield and complication rate of transbronchial lung cryobiopsy using 1.1mm probe in patients with interstitial lung disease

PLOS One

Dear Dr. Adams,

Thank you for submitting your manuscript to PLOS ONE. After careful consideration, we feel that it has merit but does not fully meet PLOS ONE’s publication criteria as it currently stands. Therefore, we invite you to submit a revised version of the manuscript that addresses the points raised during the review process.

Please submit your revised manuscript by Aug 01 2026 11:59PM. If you will need more time than this to complete your revisions, please reply to this message or contact the journal office at plosone@plos.org. When you're ready to submit your revision, log on to https://www.editorialmanager.com/pone/ and select the 'Submissions Needing Revision' folder to locate your manuscript file.

Please include the following items when submitting your revised manuscript:

- A letter that responds to each point raised by the academic editor and reviewer(s). You should upload this letter as a separate file labeled 'Response to Reviewers'.
- A marked-up copy of your manuscript that highlights changes made to the original version. You should upload this as a separate file labeled 'Revised Manuscript with Track Changes'.
- An unmarked version of your revised paper without tracked changes. You should upload this as a separate file labeled 'Manuscript'.

If you would like to make changes to your financial disclosure, please include your updated statement in your cover letter. Guidelines for resubmitting your figure files are available below the reviewer comments at the end of this letter.

If applicable, we recommend that you deposit your laboratory protocols in protocols.io to enhance the reproducibility of your results. Protocols.io assigns your protocol its own identifier (DOI) so that it can be cited independently in the future. For instructions see: https://journals.plos.org/plosone/s/submission-guidelines#loc-laboratory-protocols. Additionally, PLOS ONE offers an option for publishing peer-reviewed Lab Protocol articles, which describe protocols hosted on protocols.io. Read more information on sharing protocols at https://plos.org/protocols?utm_medium=editorial-email&utm_source=authorletters&utm_campaign=protocols.

As the corresponding author, your ORCID iD is verified in the submission system and will appear in the published article. PLOS supports the use of ORCID, and we encourage all coauthors to register for an ORCID iD and use it as well. Please encourage your coauthors to verify their ORCID iD within the submission system before final acceptance, as unverified ORCID iDs will not appear in the published article. *Only*  the individual author can complete the verification step; PLOS staff *cannot*  verify ORCID iDs on behalf of authors.

We look forward to receiving your revised manuscript.

Kind regards,

Kartikeya Rajdev, MD

Academic Editor

PLOS One

Journal Requirements:

If the reviewer comments include a recommendation to cite specific previously published works, please review and evaluate these publications to determine whether they are relevant and should be cited. There is no requirement to cite these works unless the editor has indicated otherwise.

Please review your reference list to ensure that it is complete and correct. If you have cited papers that have been retracted, please include the rationale for doing so in the manuscript text, or remove these references and replace them with relevant current references. Any changes to the reference list should be mentioned in the rebuttal letter that accompanies your revised manuscript. If you need to cite a retracted article, indicate the article’s retracted status in the References list and also include a citation and full reference for the retraction notice.

[Note: HTML markup is below. Please do not edit.]

Reviewers' comments:

Reviewer's Responses to Questions

**Comments to the Author**

1. If the authors have adequately addressed your comments raised in a previous round of review and you feel that this manuscript is now acceptable for publication, you may indicate that here to bypass the “Comments to the Author” section, enter your conflict of interest statement in the “Confidential to Editor” section, and submit your "Accept" recommendation.

Reviewer #1: All comments have been addressed

Reviewer #2: All comments have been addressed

Reviewer #3: All comments have been addressed

**********

2. Is the manuscript technically sound, and do the data support the conclusions?

The manuscript must describe a technically sound piece of scientific research with data that supports the conclusions. Experiments must have been conducted rigorously, with appropriate controls, replication, and sample sizes. The conclusions must be drawn appropriately based on the data presented.

Reviewer #1: Yes

Reviewer #2: Yes

Reviewer #3: Yes

**********

3. Has the statistical analysis been performed appropriately and rigorously?

Reviewer #1: Yes

Reviewer #2: Yes

Reviewer #3: Yes

**********

4. Have the authors made all data underlying the findings in their manuscript fully available?

The PLOS Data policy requires authors to make all data underlying the findings described in their manuscript fully available without restriction, with rare exception (please refer to the Data Availability Statement in the manuscript PDF file). The data should be provided as part of the manuscript or its supporting information, or deposited to a public repository. For example, in addition to summary statistics, the data points behind means, medians and variance measures should be available. If there are restrictions on publicly sharing data—e.g. participant privacy or use of data from a third party—those must be specified.

Reviewer #1: Yes

Reviewer #2: Yes

Reviewer #3: Yes

**********

5. Is the manuscript presented in an intelligible fashion and written in standard English?

PLOS ONE does not copyedit accepted manuscripts, so the language in submitted articles must be clear, correct, and unambiguous. Any typographical or grammatical errors should be corrected at revision, so please note any specific errors here.

Reviewer #1: Yes

Reviewer #2: Yes

Reviewer #3: Yes

**********

6. Review Comments to the Author

Please use the space provided to explain your answers to the questions above. You may also include additional comments for the author, including concerns about dual publication, research ethics, or publication ethics. (Please upload your review as an attachment if it exceeds 20,000 characters)

Reviewer #1: Thank you for submitting the revision. Respone to the reviewers comments is appreciated. I feel this manuscript will make a significant contribution to IP literature.

Reviewer #2: The authors have adequately addressed the major concerns raised during the prior review. The study design is now clearly described as a retrospective cohort study using a prospectively maintained registry, and the distinction between the procedure period and data collection period has been clarified.

The definition of diagnostic yield has also been clarified. The authors now explain that BAL and TBLC were considered diagnostic in 27 patients, and that all of these patients had a change in diagnosis after bronchoscopy.

The conclusions are now more appropriately cautious. The authors acknowledge that there was no direct comparison group using larger cryoprobes, surgical lung biopsy, or another control arm. This is important because the study cannot definitively prove superiority or noninferiority of the 1.1 mm probe compared with larger probes.

The added discussion about why the diagnostic yield in this study was lower than prior 1.1 mm cryoprobe studies is also useful. Differences in bronchoscopic platform, patient complexity, and definition of diagnostic yield are reasonable explanations.

The authors also corrected the apparent typographic errors in Table 5 and reviewed the odds ratios. This resolves a key presentation issue from the prior version.

Overall, this is a clinically relevant and useful study. It adds practical data on the diagnostic yield and complication rate of 1.1 mm transbronchial lung cryobiopsy using robotic bronchoscopy guidance in patients with ILD. The main limitations remain the retrospective design, small sample size, lack of a control group, and single-center experience, but these are now clearly acknowledged. The conclusions are supported by the data as revised. I believe the manuscript is suitable for publication.

Reviewer #3: - The pre-TBLC hypersensitivity pneumonitis (HP) threshold appears conservative but guideline-consistent. That is reasonable, but it likely contributes to the large apparent post-TBLC shift toward HP (since many patients with antigen exposure and suggestive features were initially classified as unclassifiable ILD until tissue sampling increased diagnostic confidence). I would encourage the authors to acknowledge that this could be an important limitation of the baseline classification scheme of HP and not imply that all post-TBLC diagnostic movement, especially in this category, reflects newly discovered disease.

- Typo TLBC was noted, please change that to TBLC

**********

7. PLOS authors have the option to publish the peer review history of their article (what does this mean?). If published, this will include your full peer review and any attached files.

If you choose “no”, your identity will remain anonymous but your review may still be made public.

**Do you want your identity to be public for this peer review?**  For information about this choice, including consent withdrawal, please see our Privacy Policy.

Reviewer #1: **Yes:** Shais Jallu

Reviewer #2: **Yes:** Abhishek Singh, MD

Reviewer #3: **Yes:** Narayana Gandham

**********

[NOTE: If reviewer comments were submitted as an attachment file, they will be attached to this email and accessible via the submission site. Please log into your account, locate the manuscript record, and check for the action link "View Attachments". If this link does not appear, there are no attachment files.]

To ensure your figures meet our technical requirements, please review our figure guidelines: https://journals.plos.org/plosone/s/figures

You may also use PLOS’s free figure tool, NAAS, to help you prepare publication quality figures: https://journals.plos.org/plosone/s/figures#loc-tools-for-figure-preparation.

NAAS will assess whether your figures meet our technical requirements by comparing each figure against our figure specifications.

# Author comment: Author response to Decision Letter 2

**DOI:** 10.1371/journal.pone.0354460.r004

17 Jun 2026

Response to reviewers

Reviewer #1: Thank you for submitting the revision. Respone to the reviewers comments is appreciated. I feel this manuscript will make a significant contribution to IP literature.

We appreciate reviewer 1 comments.

Reviewer #2: The authors have adequately addressed the major concerns raised during the prior review. The study design is now clearly described as a retrospective cohort study using a prospectively maintained registry, and the distinction between the procedure period and data collection period has been clarified.

The definition of diagnostic yield has also been clarified. The authors now explain that BAL and TBLC were considered diagnostic in 27 patients, and that all of these patients had a change in diagnosis after bronchoscopy.

The conclusions are now more appropriately cautious. The authors acknowledge that there was no direct comparison group using larger cryoprobes, surgical lung biopsy, or another control arm. This is important because the study cannot definitively prove superiority or noninferiority of the 1.1 mm probe compared with larger probes.

The added discussion about why the diagnostic yield in this study was lower than prior 1.1 mm cryoprobe studies is also useful. Differences in bronchoscopic platform, patient complexity, and definition of diagnostic yield are reasonable explanations.

The authors also corrected the apparent typographic errors in Table 5 and reviewed the odds ratios. This resolves a key presentation issue from the prior version.

Overall, this is a clinically relevant and useful study. It adds practical data on the diagnostic yield and complication rate of 1.1 mm transbronchial lung cryobiopsy using robotic bronchoscopy guidance in patients with ILD. The main limitations remain the retrospective design, small sample size, lack of a control group, and single-center experience, but these are now clearly acknowledged. The conclusions are supported by the data as revised. I believe the manuscript is suitable for publication.

We appreciate reviewer 2 comments.

Reviewer #3: - The pre-TBLC hypersensitivity pneumonitis (HP) threshold appears conservative but guideline-consistent. That is reasonable, but it likely contributes to the large apparent post-TBLC shift toward HP (since many patients with antigen exposure and suggestive features were initially classified as unclassifiable ILD until tissue sampling increased diagnostic confidence). I would encourage the authors to acknowledge that this could be an important limitation of the baseline classification scheme of HP and not imply that all post-TBLC diagnostic movement, especially in this category, reflects newly discovered disease.

We have added the following statement to the limitations section of the discussion:

“Further, while our pre-TBLC diagnosis of HP was consistent with current guidelines, the diagnostic confidence of HP without BAL and TBLC may vary across centers or with future iterations of the guidelines, thus limiting the generalizability of the study.”

- Typo TLBC was noted, please change that to TBLC

We have corrected the typo

Attachment Submitted filename: R2 response to reviewers.docx

# Editor report: Decision Letter 2

**DOI:** 10.1371/journal.pone.0354460.r005

**Authors:** Kartikeya Rajdev

8 Jul 2026

Diagnostic yield and complication rate of transbronchial lung cryobiopsy using 1.1mm probe in patients with interstitial lung disease

PONE-D-26-14642R2

Dear Dr. Adams,

We’re pleased to inform you that your manuscript has been judged scientifically suitable for publication and will be formally accepted for publication once it meets all outstanding technical requirements.

Within one week, you’ll receive an e-mail detailing the required amendments. When these have been addressed, you’ll receive a formal acceptance letter and your manuscript will be scheduled for publication.

An invoice will be generated when your article is formally accepted. Please note, if your institution has a publishing partnership with PLOS and your article meets the relevant criteria, all or part of your publication costs will be covered. Please make sure your user information is up-to-date by logging into Editorial Manager at Editorial Manager® and clicking the ‘Update My Information' link at the top of the page. For questions related to billing, please contact billing support.

If your institution or institutions have a press office, please notify them about your upcoming paper to help maximize its impact. If they’ll be preparing press materials, please inform our press team as soon as possible -- no later than 48 hours after receiving the formal acceptance. Your manuscript will remain under strict press embargo until 2 pm Eastern Time on the date of publication. For more information, please contact onepress@plos.org.

Kind regards,

Kartikeya Rajdev, MD

Academic Editor

PLOS One

Additional Editor Comments (optional):

Reviewers' comments:

# Editor report: Acceptance letter

**DOI:** 10.1371/journal.pone.0354460.r006

**Authors:** Kartikeya Rajdev

PONE-D-26-14642R2

PLOS One

Dear Dr. Adams,

I'm pleased to inform you that your manuscript has been deemed suitable for publication in PLOS One. Congratulations! Your manuscript is now being handed over to our production team.

At this stage, our production department will prepare your paper for publication. This includes ensuring the following:

* All references, tables, and figures are properly cited

* All relevant supporting information is included in the manuscript submission,

* There are no issues that prevent the paper from being properly typeset

You will receive further instructions from the production team, including instructions on how to review your proof when it is ready. Please keep in mind that we are working through a large volume of accepted articles, so please give us a few days to review your paper and let you know the next and final steps.

Lastly, if your institution or institutions have a press office, please let them know about your upcoming paper now to help maximize its impact. If they'll be preparing press materials, please inform our press team within the next 48 hours. Your manuscript will remain under strict press embargo until 2 pm Eastern Time on the date of publication. For more information, please contact onepress@plos.org.

You will receive an invoice from PLOS for your publication fee after your manuscript has reached the completed accept phase. If you receive an email requesting payment before acceptance or for any other service, this may be a phishing scheme. Learn how to identify phishing emails and protect your accounts at https://explore.plos.org/phishing.

If we can help with anything else, please email us at customercare@plos.org.

Thank you for submitting your work to PLOS ONE and supporting open access.

Kind regards,

PLOS ONE Editorial Office Staff

on behalf of

Dr. Kartikeya Rajdev

Academic Editor

PLOS One
